# The chloroplast genome of the *Peltigera elisabethae* photobiont *Chloroidium* sp. W5 and its phylogenetic implications

**DOI:** 10.3389/fgene.2025.1602048

**Published:** 2025-07-23

**Authors:** Guldiyar Adil, Shenglei Liu, Xiaoyan Bao, Reyim Mamut

**Affiliations:** College of Life Sciences and Technology, Xinjiang University, Urumchi, Xinjiang, China

**Keywords:** lichen symbiosis, chloroplast genome, repeat sequences, genomics, phylogeny

## Abstract

**Introduction:**

Lichens are globally distributed symbiotic organisms comprising fungi (mycobionts) and photosynthetic partners (photobionts), with exceptional adaptability to extreme environments. Despite growing interest in lichen symbiosis, chloroplast genome data for photobionts remain scarce, hindering insights into symbiotic coevolution and genomic architecture.

**Methods:**

To address this gap, we characterized the chloroplast genome of Chloroidium sp. W5, a photobiont of the lichen Peltigera elisabethae, using next-generation sequencing. The circular genome (190,579 bp) was assembled and annotated using a combination of bioinformatics tools, including GetOrganelle for genome assembly and GeSeq for annotation. We conducted a comprehensive analysis of the genome’s structure, gene content, and repetitive elements. Codon usage patterns were assessed using MEGA 11, and phylogenetic relationships were inferred using maximum likelihood analysis with IQ-tree.

**Results:**

The circular genome (190,579 bp) lacks the canonical quadripartite structure (LSC/IR/SSC) and exhibits a strong AT bias (56.1%). Annotation identified 110 functional genes, including 79 protein-coding genes, 28 tRNAs, and 3 rRNAs. Repetitive sequence analysis revealed 5,000 dispersed repeats (2.62% of the genome), predominantly forward and palindromic types, with SSR loci showing a significant A/T preference. Codon usage analysis demonstrated a pronounced bias toward A/U-ending codons (RSCU > 1), suggesting translational adaptation to symbiotic nutrient constraints. Phylogenetic reconstruction robustly placed Chloroidium sp. W5 within the Watanabeales clade (ML = 100), while synteny analysis revealed extensive genomic rearrangements compared to close relatives.

**Discussion:**

These findings enrich the chloroplast genome database for lichen photobionts, shedding light on symbiosis-driven genomic plasticity and providing a foundation for studying host-photobiont coevolution and lichen ecological adaptation.

## Introduction

Lichens represent a paradigmatic case of symbiosis involving heterotrophic fungi (mycobionts) and a population of compatible photoautotrophic microorganisms (photobionts), such as algae (phycobionts) and/or cyanobacteria (cyanobionts) (Garrido-Benavent and Pérez-Ortega, 2017). They are extremely ecologically adaptable and widespread in extreme environments such as deserts, polar regions and high mountains (Nash, 1996). As ‘pioneer species’ in the ecosystem, lichens play an irreplaceable role in soil formation, carbon and nitrogen cycling, and biogeochemical processes (Honegger, 2001). Historically viewed as rigid binary partnerships between fungi and photosynthetic organisms (algae/cyanobacteria) (Schwendener, 1869), lichens are now understood to thrive through dynamic, diverse symbioses rather than fixed species-specific interactions. Lichen-forming fungi have the capacity to bind to a broad spectrum of genotypes or species of photosynthetic partners, thus deviating from established binding patterns (Allen and Lendemer, 2022). Lichen algae may look and behave quite differently in symbiosis with different lichen-forming fungi, in the free-living condition in nature and in aposymbiotic laboratory culture (Ahmadjian, 1967; Bubrick, 1988; Sanders and Masumoto, 2021). All this has hindered progress in clarifying their identities, phylogenies and life histories. Schwendener (1869) was the first to survey lichen ‘gonidia’ in a phycological context, recognizing them as organisms distinct from the surrounding fungus that correspond to known taxa of free living algae. Although there has been much research on the genetic characteristics of symbiotic fungi (Grube and Berg, 2009; Resl et al., 2022; Cometto et al., 2024), the composition and evolutionary mechanism of the chloroplast genome of symbiotic algae is still poorly understood (Spribille et al., 2022), which severely limits the in-depth analysis of lichen taxonomic relationships, host-symbiont co-evolution and ecological adaptation mechanisms.

The chloroplast genome (cpDNA), a circular double-stranded DNA molecule maintaining autonomous replication within plastids, contains genes critical for photosynthesis and organellar gene expression. Its structural evolution - particularly in gene content, repetitive elements, and nucleotide composition - reflects adaptive responses to environmental pressures (Howe et al., 2003; Spribille et al., 2022). Green algae (divisions Charophyta and Chlorophyta) serve as essential photosynthetic partners in lichen symbioses. Among these, members of the Trebouxiophyceae, Ulvophyceae, and Chlorophyceae classes (Chlorophyta) are particularly prominent as photobionts (Leliaert et al., 2012). Molecular data indicate the presence of numerous putative cryptic species within the green algae genus (e.g., approximately 30 cryptosporidia have been identified within *Trebouxia*, far exceeding traditional classifications) (Leavitt et al., 2015). A significant impediment to the advancement of lichen symbiotic algae research is the significantly constrained availability of axenic cultures for study, a constraint that stems primarily from the technical challenges in isolating and culturing these organisms, which has resulted in an extremely limited pool of viable research strains (Muggia et al., 2020). The existing genome sequencing efforts for symbiotic algae show pronounced taxonomic skewness, with overrepresentation of model species like *Trebouxia* sp. DW1 (Wang et al., 2022) at the expense of broader phylogenetic coverage. This limitation becomes particularly salient given recent molecular evidence uncovering widespread cryptic diversity in chlorophytic symbionts. In *Trebouxia* alone, phylogenetic analyses have detected around 30 evolutionarily distinct lineages that defy differentiation through classical taxonomic criteria (Leavitt et al., 2015), suggesting current biodiversity assessments may significantly underestimate true species richness. Genomic analyses of individual strains enable precise delineation of genetic boundaries, thus confirming the independent evolutionary origins of *Asterochloris* and *Trebouxia*. This provides molecular evidence for revising the classification system of lichen photosynthetic symbionts and resolving long-standing morphological confusions (Sanders and Masumoto, 2021). Recent studies have revealed that lichen symbiotic algal plastid genome evolution is characterised by significant symbiosis specificity. In considering the Trebouxiophyceae taxon, Puginier et al. (2024) determined that symbiotic algae have acquired the glycoside hydrolase 8 (GH8) gene via horizontal gene transfer (HGT). This gene encodes an enzyme capable of specifically degrading β-1,3/1,4-glucans (e.g., lichenin) in the cell walls of lichen-fungal symbionts (LFS). This molecular mechanism directly contributes to the formation of the symbiotic interface. It is imperative to note that Iha et al. (2021) demonstrated that the chloroplast genome of the lichen symbiotic microalgae *Trebouxia* exhibits distinctive features of structural remodelling. Specifically, its inverted repeat sequences (IRs) undergo a significant shortening, while key ribosomal protein genes (e.g., *rps4*) shift to the nuclear genome. This reorganisation of gene functions may enhance symbiotic adaptation by optimising nucleoplasmic co-regulatory mechanisms.

In a comparative genomics study, Lemieux et al. (2014) found that *Coccomyxa subellipsoidea* in the free-living state maintains an intact quadripartite plastid genome structure (IR/LSC/SSC), with a genome size of 160–180 kb encoding about 100–110 genes, including a complete cluster of photosynthesis-related genes (PSA, PSB, etc.), ribosomal RNA (rrn) and transfer RNA (trn) genes. In contrast, the symbiotic *Coccomyxa viridis* studied by Muggia et al. (2020) exhibited partial or complete loss of the IR region, a phenomenon that may be related to genomic reduction due to symbiotic selection pressure exerted by the host fungus. A comparative analysis by Wang et al. (2022) revealed that the *Coccomyxa* chloroplast genome exhibited a higher functional gene conservation, particularly concerning key functional genes involved in carbon fixation (*rbcL*) and photosystem II assembly (*psbA*), in comparison to the symbiotic *Trebouxia*. This observation was corroborated by Sanders and Masumoto (2021). This discrepancy may be indicative of the distinct metabolic plasticity exhibited by *Coccomyxa* in its free-living and symbiotic states.

*Peltigera* Willd. is one of the earliest lichen genera described (Willdenow, 1787). Subsequent studies have shown that there are two different types of symbionts within the *Peltigera*: (1) a binary symbiosis consisting of a *cyanobacterium* (e.g., *Nostoc*) and a fungus (2) a green alga (e.g., *Coccomyxa*); as the main photosynthetic symbiont (Miadlikowska et al., 2000). Previous research has mainly focused on traditional taxonomy, mitochondrial genome analysis and biological activity and component synergy (Wei et al., 2009). Traditional classification of lichen symbionts has primarily relied on morphological characteristics and short molecular markers (e.g., ITS, *rbcL*) (Howe et al., 2003; Wei et al., 2009; Guo et al., 2021; Resl et al., 2022; Spribille et al., 2022; Pushpavathi and Krishnamurthy, 2024; Cometto et al., 2024; Li et al., 2025a). However, these approaches frequently result in ambiguous species delimitation due to phenotypic plasticity and high sequence conservation among closely related taxa (Armaleo et al., 2019). Recent advances in genomic sequencing have provided new taxonomic insights, exemplified by the complete chloroplast DNA (cpDNA) assembly of *Trebouxia* sp. TR9, isolated from the lichen *Ramalina farinacea* (Martínez-Alberola et al., 2020). Comparative genomic analyses of this strain with other Trebouxiophyceae species have demonstrated the utility of whole-organelle genomes in refining lichen systematics. Building upon these findings, our laboratory successfully sequenced and assembled the complete cpDNA of *Trebouxia* sp. DW1, a photobiont isolated from *Peltigera rufescens*, and conducted comparative genomic analyses with related Trebouxiophyceae species (Wang et al., 2022).

In this study, the green alga symbiont (*Chloroidium* sp. W5) of *P. elisabethae* was selected as the study subject, and the following work was systematically carried out: (1) Chloroplast genome composition analysis: high-throughput sequencing and comparative genomics were used to reveal the structural characteristics of the symbiotic algae chloroplast genome (such as gene content, repetitive sequence distribution and GC content) and to identify specific variations driven by the symbiotic environment. (2) Repetitive sequence analysis: This analysis reveals the dynamics of the *Chloroidium* sp. W5 genome structure and provides important clues for studying coevolutionary mechanisms in lichen symbiotic systems. (3) Codon preference analysis: The codon usage preference of *Chloroidium* sp. W5 was calculated. By analysing the frequency of codon usage, it reflects the evolutionary pressure and adaptive changes of the chloroplast genome. (4) Synteny Analysis: Revealing the conserved and dynamically evolving features of *Chloroidium* sp. W5 genomic structure and emphasizing the relevance of its structural rearrangement to symbiotic adaptation through synteny analysis. (5) Phylogenetic reconstruction and taxonomic determination: A highly supported phylogenetic tree was reconstructed using both whole-genome and chloroplast genomic data from 18 Trebouxiophycean species. Here, we discuss the structure, organization, gene content of *Chloroidium*, a common terrestrial coccoid green alga, and comparison analysis with other chloroplast genomes reported for Trebouxiophyceae. We also provide a fairly resolved phylogenetic reconstruction on the basis of well-conserved chloroplast genes coding for proteins. It is evident that the study methodology can be applied to non-model lichen symbionts.

## Materials and methods

### Sample collection and identification

In this study, specimens of *Peltigera elisabethae* Gyeln. were collected from Xinjiang, Northwest China. Detailed species information is provided in Supplementary Table 1. All voucher specimens were deposited in the Herbarium of the College of Life Science and Technology, Xinjiang University (XJU). Species identification was conducted using an integrative approach combining morphological, anatomical, and chemical analyses: (1) Thallus shape, color, and upper surface texture were examined under a dissecting microscope. Specialized structures—including cephalodia (soredia, isidia, tomentum), vein morphology (cephalodia, isidia, sorelia, tomentum, rhizine characteristics), and apothecial features (size, color, shape)— were recorded. (2) Photobiont analysis: The photobiont type (green algae or cyanobacteria), distribution, cell morphology, and dimensions were observed using a stereoscopic microscope.

### Phycobiont isolation and culture conditions

Three replicate samples (ca. 1 cm^2^) were collected from a single *P*. *elisabethae* specimen. Each sample was processed as follows: (1) rehydration in sterile water for ≥30 min, (2) surface sterilization through 2-3 sterile water rinses, and (3) homogenization in 1,000 μL sterile water using an autoclaved mortar in a laminar flow hood until complete fragmentation. Microscopic examination (10 μL aliquot) confirmed algal cell debris presence. Approximately 50–100 μL of homogenate was cultured on BGII solid medium (Hopebio HB8793) via spread-plate method under controlled conditions (20°C, 12/12 h light/dark cycle, 3,000 lux illumination) using Illuminated Incubator (Ningbo Jiangnan Instrument Factory, model RXZ-436). Initial microcolonies emerged after 10 days, developing visible colonies within 15–20 days. Pure cultures were obtained through successive streak-plate isolation on BGII medium. The isolated *Chloroidium* sp. W5 strains exhibited slow growth, requiring 30–40 days cultivation for experimental use, with concurrent strain preservation. Cultivation maintained the original light regime and medium composition throughout subculturing. Whole-genome sequencing (WGS) of the purified target strain *Chloroidium* sp. W5 was subsequently performed using the DNBSEQ sequencing platform (Shenzhen, China).

### Chloroplast genome assembly and annotation

The chloroplast genome of *Chloroidium* sp. W5 was assembled using GetOrganelle V1.7.4.1 (Jin et al., 2020) followed by genome annotation using the web-based platform GeSeq V2.03 (Tillich et al., 2017) with default parameters. The annotation results were manually refined to verify gene boundaries, intron-exon structures, and functional assignments using Geneious V2022.1.1 (Kearse et al., 2012). A circular genome map was generated using OGDRAW V1.3.1 (Lohse et al., 2007) (https://chlorobox.mpimp-golm.mpg.de/OGDraw.html), and further optimized for visual clarity using vector graphic editing software.

### Repetitive sequence analysis

This study characterized repetitive elements in the *Chloroidium* sp. W5, focusing on four distinct categories: interspersed repeats, tandem repeats, simple sequence repeats (SSRs), and dispersed duplications. Tandem repeat detection was performed through Tandem Repeats Finder V4.09 (Benson, 1999) using parameters optimized for plastid genomes: alignment weights 2/7/7 (match/mismatch/indel) and minimum alignment score 50. Interspersed repeats were identified using REPuter (Kurtz et al., 2001) with Hamming distance 3, maximum length 5,000 bp, and minimum size 30 bp. SSRs were detected through MISA V2.1 (Beier et al., 2017) with motif thresholds set as follows: mono- (≥10), di- (≥5), tri- (≥4), tetra- (≥3), penta- (≥3), and hexanucleotide (≥3) repeats. All identified repeats were mapped to coding and non-coding regions using TBtools V2.018 (Chen et al., 2020) with manual verification of repeat boundaries against annotated gene features.

### Codon usage analysis

Codon usage patterns and interspecific divergence in *Chloroidium* sp. W5 chloroplast genomes were analyzed through synonymous codon preference assessment of conserved CDSs. The CDSs were systematically extracted using PhyloSuite V1.2.2 (Zhang et al., 2020) with stringent filtering including removal of pseudogenes and truncated ORFs, exclusion of RNA-coding genes, and retention of sequences ≥300 bp to ensure statistical reliability. Relative synonymous codon usage (RSCU) values were then calculated in MEGA 11 (Tamura et al., 2021) following standard codon normalization protocols.

### Synteny analysis

The *Chloroidium* sp. W5 chloroplast genome obtained in this study was analysed for covariance with three chloroplast genomes from the family Coleoptera (*Chloroidium* sp.; *Polulichloris maxima*; *Kalinella pachyderma*). The covariance analysis was performed using Mauve V2.4.0 (Darling et al., 2004).

### Phylogenetic analysis

Phylogenetic analysis was performed using *Chloroidium* sp. W5 and 18 related sequences from GenBank (Supplementary Table 2), with *Schizomeris leibleinii* and *Stigeoclonium helveticum* as outgroups. PCGs were extracted using Phylosuite V1.2.3 (Zhang et al., 2020), aligned with MAFFT V7.475 (Katoh et al., 2019), and concatenated using Sequence Matrix (Vaidya et al., 2011). The optimal evolutionary model was determined, and maximum likelihood (ML) analysis was conducted with IQ-tree V1.6.8 (Nguyen et al., 2015). The resulting phylogenetic tree was visualized using FigTree V1.4.3.

## Results

### Features of the newly assembled *Chloroidium* sp. W5 chloroplast genome

**FIGURE 1:**
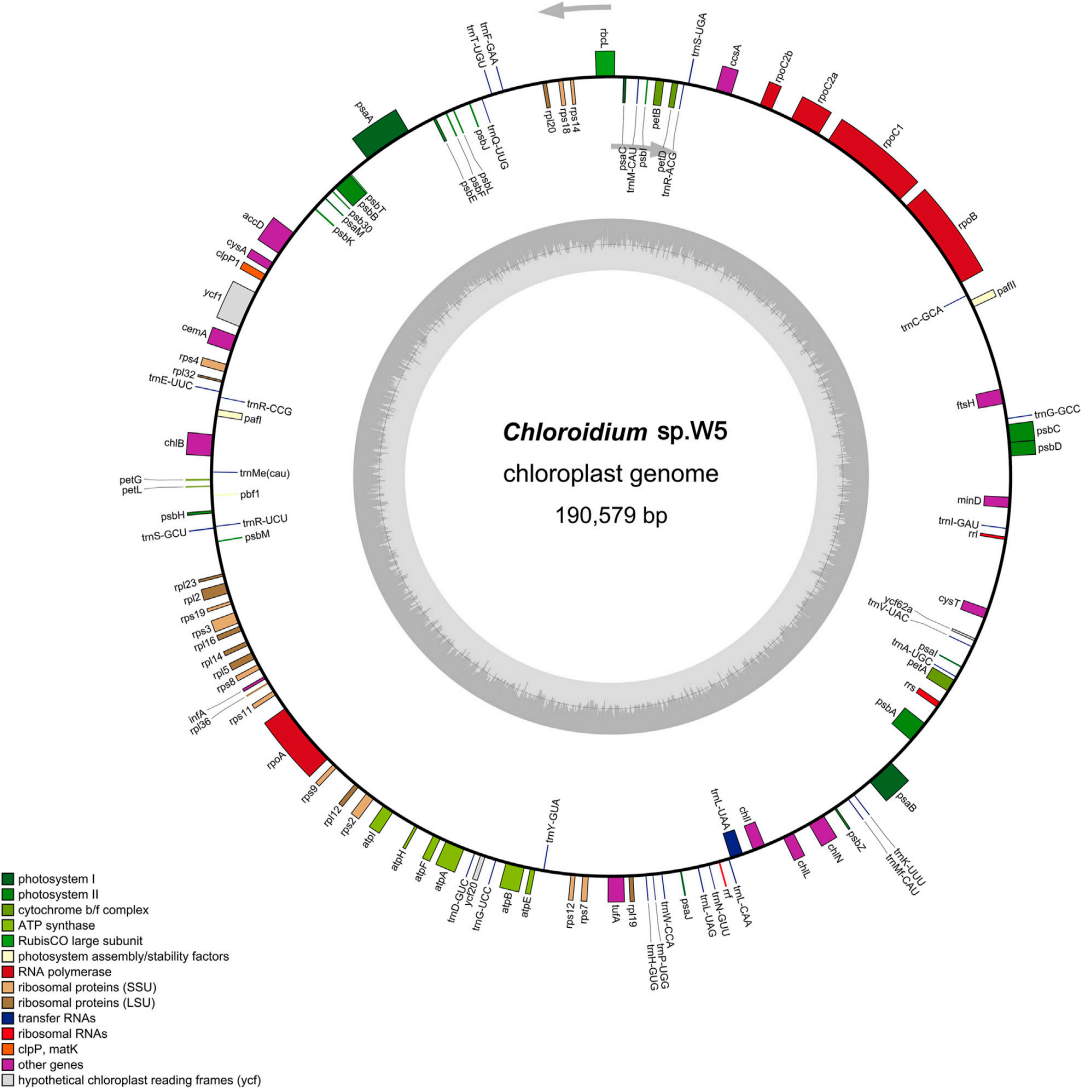
Circular maps of *Chloroidium* sp. W5 chloroplast genome. Genes with different functions are represented by different colors. The genes inside the circle are on the direct strand, and the genes outside the circle are on the reverse strand.

The complete chloroplast genome sequence of *Chloroidium* sp. W5 has been deposited in GenBank under accession number PV414516. This circular DNA molecule measures 190,579 bp in length (Figure 1), lacking the typical quadripartite chloroplast structure characterized by the absence of a large single copy region (LSC), small single copy region (SSC), and inverted repeat regions (IR). The nucleotide composition of the genome is as follows: A 53,653 (28.1%), C 41,483 (21.8%), G 42,288 (22.2%), and T 53,155 (27.9%). A total of 110 coding genes were annotated in the chloroplast genome, including 79 protein-coding genes, 28 tRNA genes, and 3 rRNA genes (Supplementary Table 3).

### Repetitive element analysis

**FIGURE 2:**
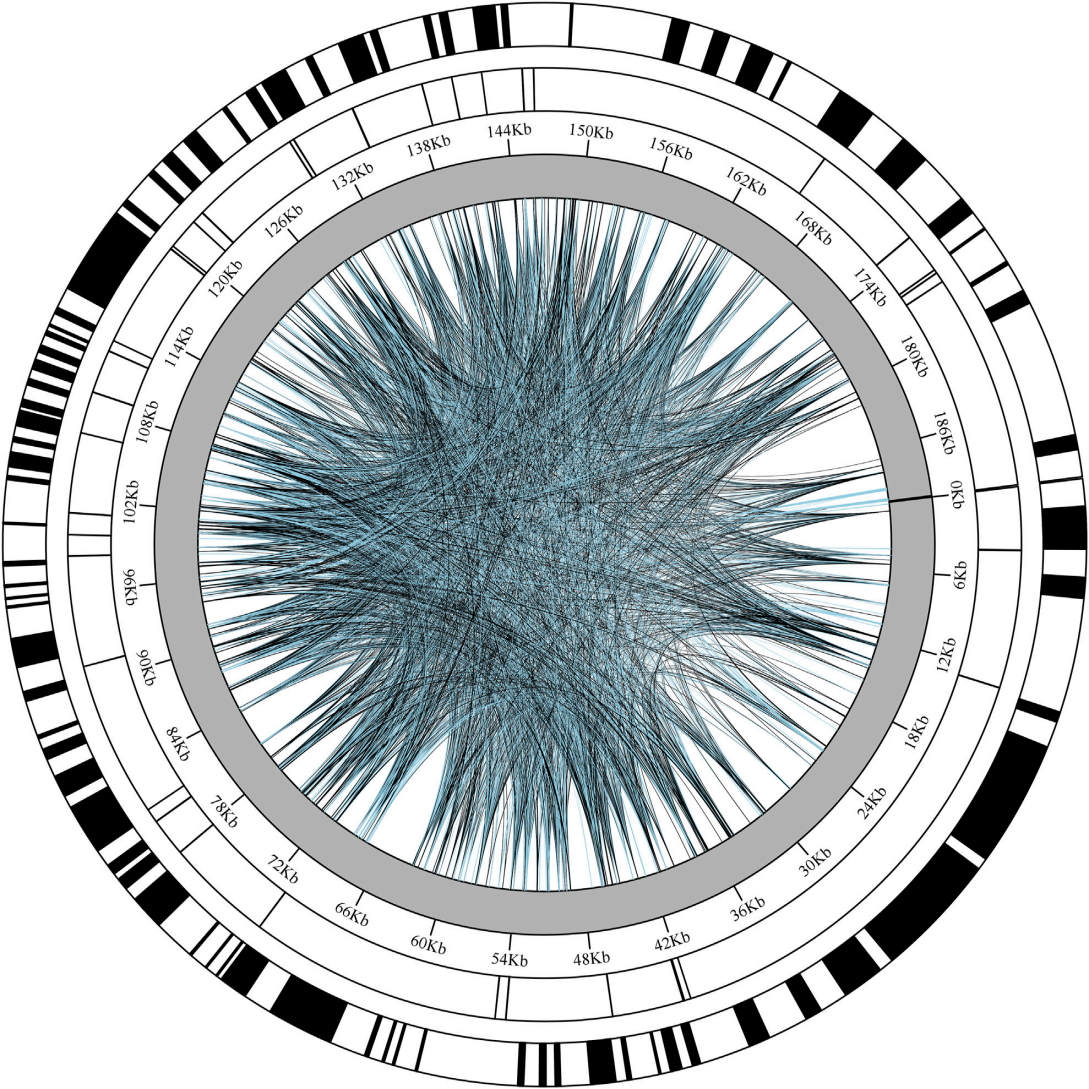
The repeated distribution map of *Chloroidium* sp. W5 chloroplast genome. Each circle from inside to outside represents: Interspersed repeats (black represents Forward, blue represents Palindromic); SSRs; Tandem repeats.

A systematic analysis of repeat sequences in the chloroplast genome of *Chloroidium* sp. W5 (Figure 2) revealed significant characteristics of dispersed repeats. A total of 5,000 dispersed repeats were identified, accounting for 2.62% of the total genome length. The length distribution of these repeats spanned a wide range, from 98 bp to 190,313 bp, with the longest repeats located in two regions: 189,385 bp–189,650 bp (265 bp) and 190,313 bp–190,578 bp (265 bp). In terms of repeat type distribution, forward repeats (F, 2,448) and palindromic repeats (P, 2,552) were the predominant forms, while complementary repeats (C) and reverse repeats (R) were not detected. Analysis of SSRs using MISA software identified 40 SSRs loci in the genome, with lengths ranging from 10 to 20 bp. Among these, mononucleotide repeats were the most abundant (22, 55%), followed by hexanucleotide repeats (6), while tri-, tetra-, and pentanucleotide repeats were each detected twice (2). Combined with the genomic base composition characteristics (Supplementary Tables 4, 5), the chloroplast genome of *Chloroidium* sp. W5 exhibited a significantly higher AT content compared to GC content, which explains the pronounced preference for A/T bases in SSR loci. Further analysis of tandem repeats identified 24 tandem repeat loci in the *Chloroidium* sp. W5 genome. Notably, significant T-base enrichment was observed in specific regions, such as 49,182 bp–49,218 bp (72% T content) and 124,167 bp–124,197 bp (51% T content).

### Codon usage analysis

**FIGURE 3:**
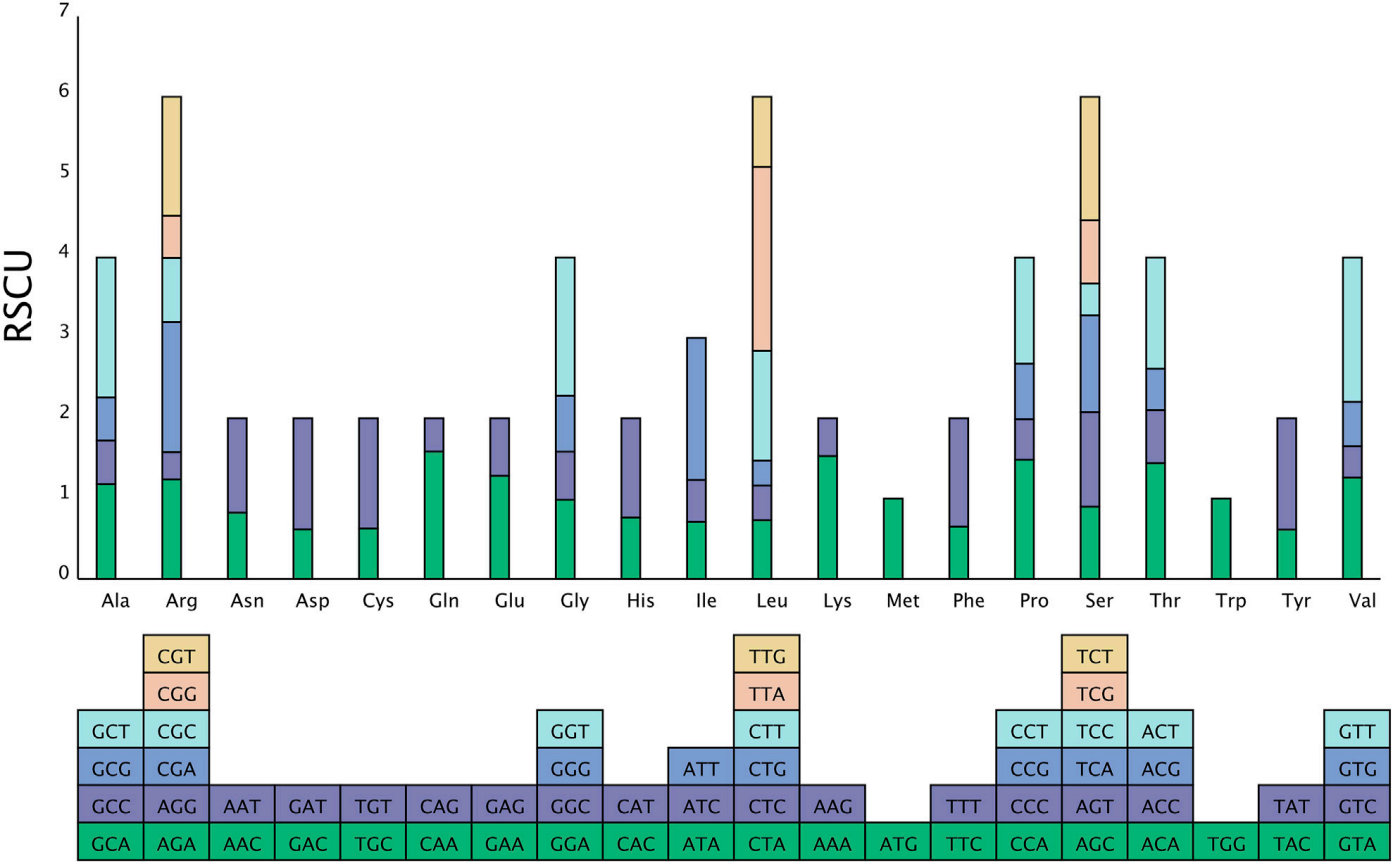
Codon usage analysis of *Chloroidium* sp. W5 chloroplast genome. The X-axis comprises the 20 standard amino acids that encode the protein, and the encoding codon is featured below each amino acid. The Y-axis is the frequency of codon usage.

Analysis of the relative synonymous codon usage (RSCU) across the entire chloroplast genome of *Chloroidium* sp. W5 (Figure 3) revealed that the coding sequences comprise 64 codons, encoding 20 amino acids. Codon usage analysis indicated that leucine (Leu), serine (Ser), and arginine (Arg) are each encoded by six codons, whereas methionine (Met) and tryptophan (Trp) are encoded by only one codon. Among these, the codon UUA, encoding leucine (Leu), exhibited the highest usage frequency. Within the *Chloroidium* sp. W5 chloroplast genome, 28 codons had an RSCU value ≥ 1, all of which ended with A/U, demonstrating a pronounced A/U bias (Supplementary Table 6).

### Synteny analysis

**FIGURE 4:**
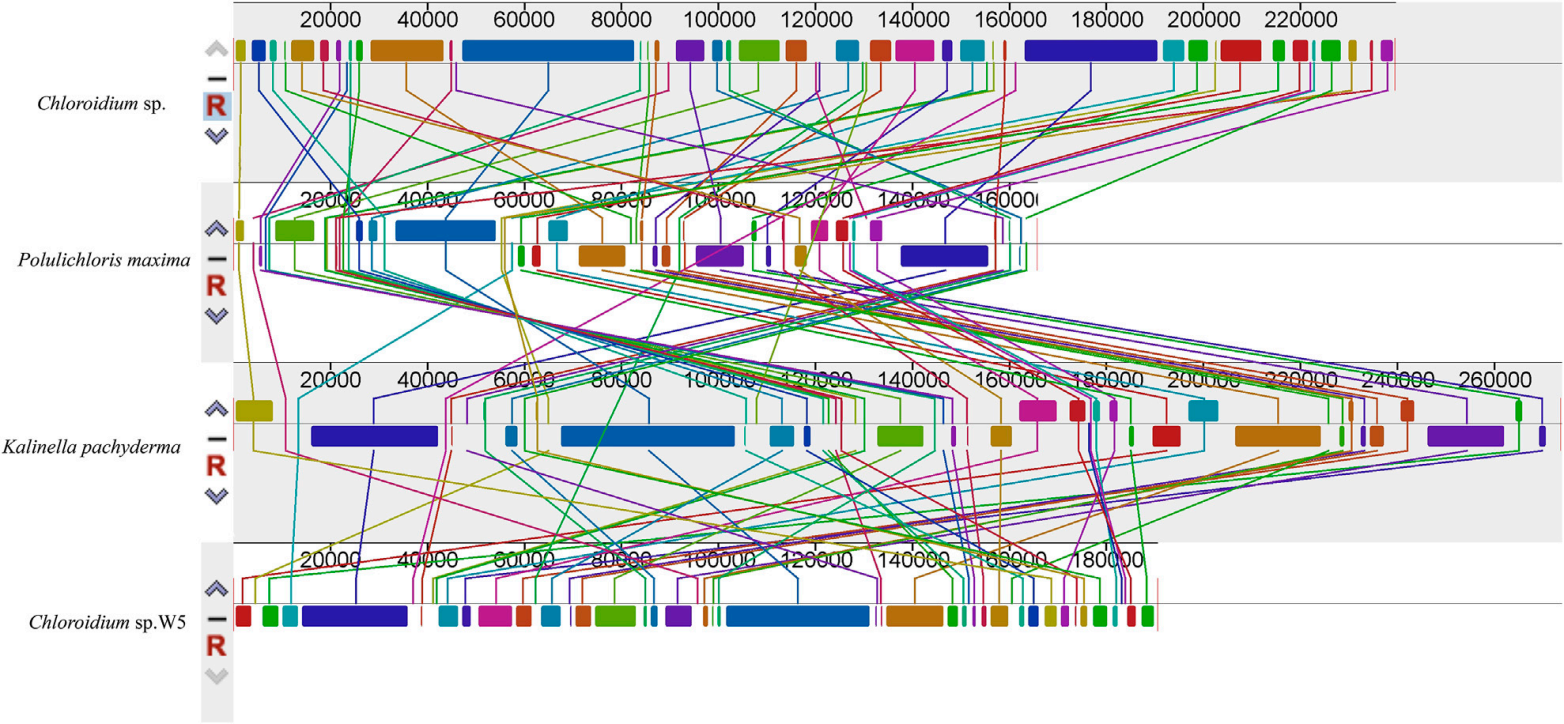
Comparative chloroplast genome rearrangement analysis of the 4 Watanabeales species (*Chloroidium* sp.; *Polulichloris maxima*; *Kalinella pachyderma*; *Chloroidium* sp. W5) using Mauve. Homologous regions between different species were represented by the same color blocks. Species in this study are shown in bold.

Colinearity analysis (Figure 4) reveals the degree of conservation and dynamic evolution of chromosome structure during species evolution by systematically comparing the linear arrangement characteristics of homologous sequences between genomes of different species. In the visualisation of covariance mapping, different colour blocks usually correspond to specific nucleotide conserved regions or amino acid functional domains, and the change in their colour gradient intuitively reflects the degree of sequence homology attenuation. It is noteworthy that the genomic covariance pattern of *Chloroidium* sp. W5 shows a remarkable specificity: The homologous regions of this species not only frequently break and recombine, but also form a highly complex network of topological connectivity with neighbouring species. This disordered covariance suggests that the genome may have undergone large-scale structural remodelling during its evolutionary history, including but not limited to asymmetric insertions/deletions of chromosomal segments, multilocus inversions and transchromosomal translocations. These cumulative genomic changes led to significant divergence in gene arrangement. This provides molecular evidence for its unique evolutionary pathway.

### Phylogenetic analysis

**FIGURE 5:**
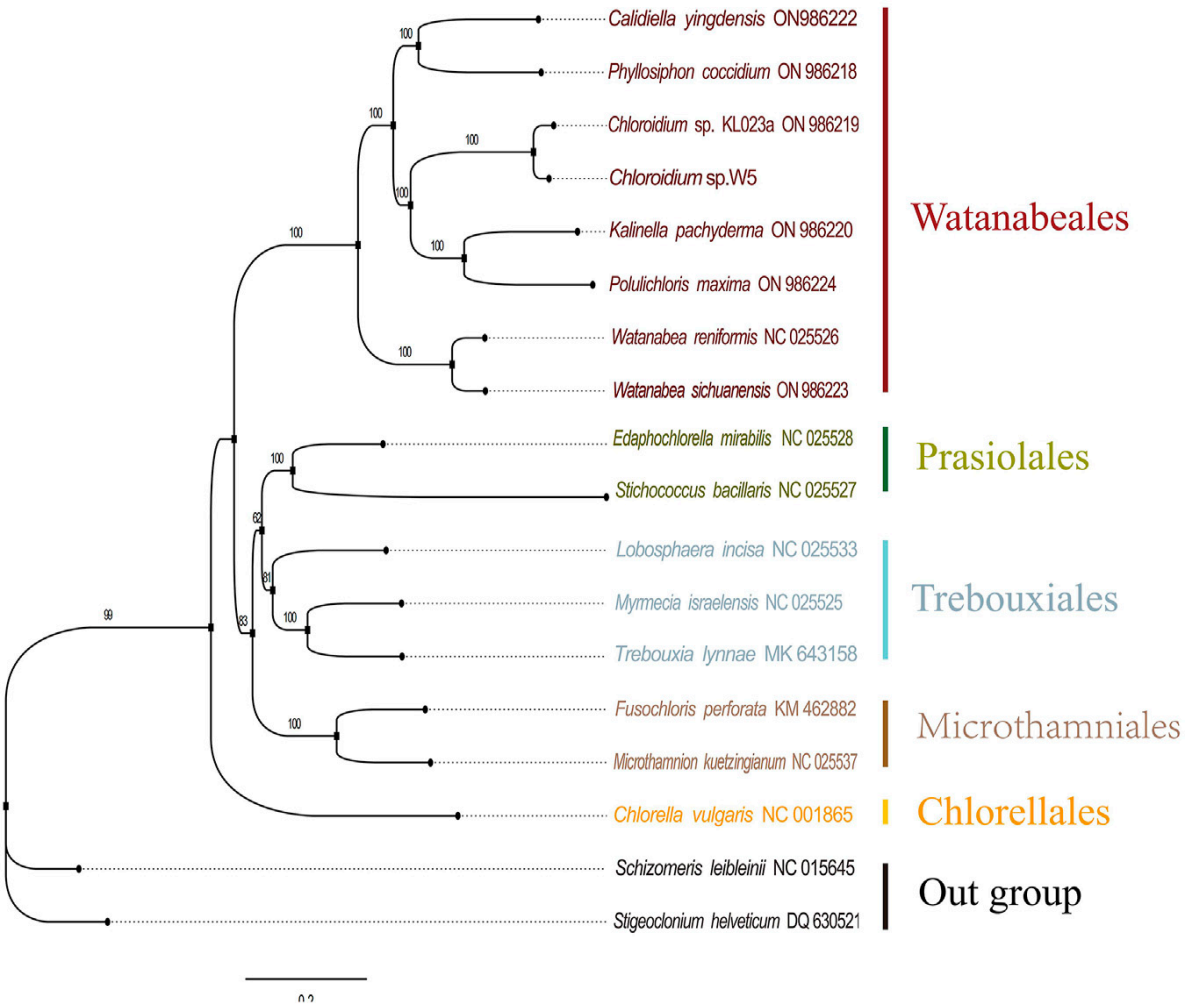
The phylogenetic tree of 18 species of Trebouxiophyceae and Chlorophyceae based on the PCGs.

Phylogenetic analysis of the chloroplast genome sequences was performed using the maximum likelihood method (Figure 5), which demonstrated that *Chloroidium* sp. W5 forms a highly supported sister branch (ML = 100) with the congeneric species *Chloroidium* sp. This finding is consistent with the results of previous phylogenetic studies based on multiple loci (Darienko et al., 2015), thereby providing further confirmation of the taxonomic status of the strain. The construction of a phylogenetic tree reveals that species belonging to the order Watanabeales form a monophyletic group (ML = 100), thereby substantiating the phylogenetic independence of this taxonomic group. It is noteworthy that *Chloroidium* sp. W5 branches farther away from *Chlorella vulgaris* and has significant phylogenetic isolation (ML = 99), a result that is consistent with recent studies on the revision of the phylogenetic classification of the family Chlorellaceae (Bock et al., 2011), confirming that the two belong to different taxonomic units. Furthermore, the phylogenetic topology demonstrates a moderately supported phylogenetic relationship between the Prasiolales + Trebouxiales branch and the order Microthamniales (ML = 83), suggesting that these taxa may share a common evolutionary origin. This finding, which indicates a common evolutionary origin for these taxa, echoes the results of recent molecular clock studies on the differentiation of early green algae (Fučíková et al., 2014). Additionally, support values for all branch nodes in the phylogenetic tree are labeled above the corresponding branches, with bootstrap values exceeding 50%.

## Discussion

*Chloroidium* sp. W5, the photosynthetic symbiont of the lichen *P*. *elisabethae*, exhibits several remarkable chloroplast genomic features that provide novel insights into the evolutionary adaptation mechanisms of lichenized algae. The absence of the canonical quadripartite structure (LSC/SSC/IR) in this chloroplast genome is particularly noteworthy. This structural simplification aligns with observations in other symbiotic algae such as *Trebouxia* and *Coccomyxa* (Poquita-Du et al., 2024), suggesting potential adaptive advantages in symbiotic systems. The loss of inverted repeats (IRs), known to maintain chloroplast genome stability (Turmel et al., 2017), may indicate relaxed selective pressures in the protected symbiotic environment. Similar genome reduction patterns have been documented in other obligate symbionts (Smith and Keeling, 2015), supporting the hypothesis that symbiotic lifestyles promote genomic streamlining. In this study, comparative genomics analysis revealed significant genomic rearrangements in the chloroplast genome of *Chloroidium* sp. W5, including multiple types of structural variants such as inversions, deletions, insertions and duplications. These rearrangement events may affect gene function through multiple mechanisms. First, structural variants may alter promoter regions, particularly due to the absence of the typical chloroplast tetrameric structure (LSC/IR/SSC) in this species, which could significantly impact transcription initiation efficiency and gene expression levels (Daniell et al., 2016). Second, gene rearrangements may lead to the loss of gene function or the acquisition of novel functions, a phenomenon previously reported in chloroplast genome studies of other plants (Jansen and Ruhlman, 2012). Notably, the present study found that the chloroplast genome of *Chloroidium* sp. W5 contains a high abundance of repetitive sequences and SSR sites. These repetitive elements may promote genomic rearrangement through homologous recombination mechanisms (Wicke et al., 2011), which in turn affects gene function. From an evolutionary perspective, the stability of the chloroplast genome is critical for maintaining the stability of endosymbiotic relationships. Our results suggest that genome rearrangements may disrupt this stability, which in turn affects the symbiotic interface between chloroplasts and host cells, particularly in terms of the efficiency of material and energy exchange (Smith and Keeling, 2015). Of particular interest, the abundance of scattered repeat sequences in the *Chloroidium* sp. W5 genome may provide a genetic basis for the adaptation of this species to extreme environments by promoting genomic variation. This finding echoes the results of Chumley et al. (2006) in Pelargonium x hortorum, suggesting that repetitive sequence-mediated genome rearrangement may be an important mechanism for plant adaptation to environmental stress. Therefore, we hypothesize that the genomic plasticity feature observed in *Chloroidium* sp. W5 may be one of the key factors for its ability to survive in extreme environments. Tuller et al. (2010) demonstrated that in systems where there is high expression, such as in bacteria and yeast, natural selection tends to optimise codon usage patterns. This means that highly expressed genes tend to use the optimal codons (especially A/U ending-type codons) that match the host tRNA library. This minimises ribosomal stalling during translation and increases the efficiency of protein synthesis. It is noteworthy that the *Chloroidium* sp. W5 chloroplast genome exhibited a significant A/U-type codon preference (RSCU > 1) in this study, a finding that is highly consistent with the theoretical predictions of Tuller et al. (2010). Moreover, this codon preference demonstrated a synergistic evolutionary pattern with the significantly high AT content (56.1%) characterising this genome, strongly suggesting that this may be a genome-level adaptive strategy developed during the long-term adaptation of the lichen symbiosis system. This adaptive evolution may be achieved through the following mechanisms: (1) optimising the translation efficiency of photosynthesis-related genes to adapt to the symbiotic environment; and (2) reducing energy consumption to cope with the nutrient-limited conditions typical of lichen symbionts. In the present study, the chloroplast genomes of *Chloroidium* sp. W5 were compared with those of extremophilic red algae. The results demonstrated structural simplification (IR loss, gene reduction), repetitive sequence expansion, and functional gene specialisation. However, the adaptive mechanisms differed between the two groups. Symbiotic algae rely on host interactions and maintain symbiotic efficiency through codon optimisation and genome plasticity. In contrast, extremophilic algae respond directly to physical stresses (e.g., retention of heat- and salt-tolerance genes). Collectively, these findings lend support to the hypothesis that environmental stresses drive adaptive genome evolution, and they provide molecular evidence for understanding the evolutionary strategies of organisms in extreme or symbiotic environments.

Phylogenetic reconstructions based on chloroplast gene sequences have contributed to resolve deep level relationships within the Trebouxiophyceae (Lemieux et al., 2014). Phylogenomic analysis definitively placed *Chloroidium* sp. W5 within the Watanabeales clade, resolving previous taxonomic uncertainties (Darienko et al., 2015). Its distant relationship with *Chlorella* species supports recent revisions in Trebouxiophyceae classification (Bock et al., 2011). The moderately supported relationship between Watanabeales and the Prasiolales + Trebouxiales clade suggests these taxa may share ancestral adaptive features for symbiotic lifestyles (Wang et al., 2022). Synteny analysis (Figure 4) revealed extensive genomic rearrangements, indicating a dynamic evolutionary history for *Chloroidium* sp. W5. These structural variations may represent adaptive responses to the symbiotic environment, with similar patterns reported during niche specialization in other algal lineages (Leliaert et al., 2012). These findings confirm that lichen symbiosis imposes unique evolutionary pressures on chloroplast genomes, driving: (i) structural simplification through gene loss and relocation; (ii) genomic plasticity via repeat expansion; and (iii) nucleotide-level adaptation in codon usage and base composition.

## Data Availability

The chloroplast genome sequence of Chloroidium sp. W5 is publicly available in the NCBI GenBank database under accession number PV414516 (https://www.ncbi.nlm.nih.gov/nuccore/PV414516.1/).
