# Supplementary material for: The chloroplast genome of the Peltigera elisabethae photobiont Chloroidium sp. W5 and its phylogenetic implications
Source: Front Genet. 2025 Jul 23;16:1602048. doi: 10.3389/fgene.2025.1602048 (PMC12325071; doi:10.3389/fgene.2025.1602048)
Supplement: Supplementary file 1 [file Table1.docx]

Supplementary Material

**Supplementary Table 1 Specimen collection information**

| Species name | Latitude | Attitude | Elvation |
| --- | --- | --- | --- |
| *Peltigera elisabethae* | 48°34'09" | 87°26'36" | 1096 m |

**Supplementary Table 2 Specimen information downloaded from NCBI**

| Species name | GenBank |
| --- | --- |
| *Phyllosiphon coccidium* | ON986218 |
| *Kalinella pachyderma* | ON986220 |
| *Chloroidium sp.* | ON986219 |
| *Watanabea sichuanensis* | ON986223 |
| *Calidiella yingdensis* | ON986222 |
| *Polulichloris maxima* | ON986224 |
| *Watanabea reniformis* | NC025526 |
| *Myrmecia israelensis* | NC025525 |
| *Lobosphaera incisa* | NC025533 |
| *Stichococcus bacillaris* | NC025527 |
| *Edaphochlorella mirabilis* | NC025528 |
| *Fusochloris perforata* | KM462882 |
| *Microthamnion kuetzingianum* | NC025537 |
| *Chlorella vulgaris* | NC001865 |
| *Trebouxia lynnae* | MK643158 |
| *Stigeoclonium helveticum* | DQ630521 |
| *Schizomeris leibleinii* | NC015645 |

**Table 3 Gene annotation of the chloroplast genome of *Chloroidium* sp. W5.**

| Category | Gene group | Gene name |
| --- | --- | --- |
| Photosynthesis | Subunits of photosystem I | *psa*A,*psa*B,*psa*C,*psa*I,*psa*J,*psa*M |
|  | Subunits of photosystem II | *psb*A,*psb*B,*psb*C,*psb*D,*psb*E,*psb*F,*psb*H,*psb*I,*psb*J,*psb*K,*psb*L,*psb*M,*psb*N,*psb*T,*psb*Z1 |
|  | Subunits of cytochrome b/f complex | *pet*A,*pet*B,*pet*D,*pet*G,*pet*L |
|  | Subunits of *atp* synthase | *atp*A,*atp*B,*atp*E,*atp*F,*atp*H,*atp*I |
|  | Large subunit of rubisco | *rbc*L |
|  | Subunits photochlorophyllide reductase | *chl*B,*chl*I,*chl*L,*chl*N |
| Self-replication | Proteins of large ribosomal subunit | *rpl*12,*rpl*14,*rpl*16,*rpl*19,*rpl*2,*rpl*20,*rpl*23,*rpl*32,*rpl*36,*rpl*5 |
|  | Proteins of small ribosomal subunit | *rps*11*,rps*12*,rps*14*,rps*18*,rps*19*,rps*2*,rps*3*,rps*4*,rps*7*,rps8,rps*9 |
|  | Subunits of RNA polymerase | *rpo*A*,rpo*B,*rpo*C1,*rpo*C2a,*rpo*C2b |
|  | Ribosomal RNAs | *rrf,rrl,rrs* |
|  | Transfer RNAs | *ttrnI-GAU,trnV-UAC,trnA-UGC,trnK-UUU,trnMf-CAU,trnL-UAA,trnL-CAA,trnN-GUU,trnL-UAG,trnW-CCA,trnP-UGG,trnH-GUG,trnY-GUA,trnG-UCC,trnD-GUC,trnS-GCU,trnR-UCU,trnMe(cau),trnR-CCG,trnE-UUC,trnQ-UUG,trnT-UGU,trnF-GAA,trnM-CAU,trnR-ACG,trnS-UGA,trnC-GCA,trnG-GCC* |
| Other genes | *atp*-dependent protease subunit P | *clp*P1 |
|  | Envelope membrane protein | *cem*A |
|  | Acetyl-CoA carboxylase | *acc*D |
|  | cytochrome synthesis gene | *ccs*A |
|  | Translation initiation factor | *inf*A |
| Category | Gene group | Gene name |
| Other genes | other | *fts*H,*min*D,*tuf*A，cysT,pbf1,pafI,cysA,pafII |
|  | Conserved hypothetical chloroplast ORF | *ycf*1*,ycf*20*,ycf*62a |

Note: * indicated that genes with one intron.

**Table 4 SSRs identified in the chloroplast genome in *Chloroidium* sp. W5**

| SSRs No. | SSRs type | SSRs | size | start | end |
| --- | --- | --- | --- | --- | --- |
| 1 | p1 | (C)16 | 16 | 4061 | 4076 |
| 2 | p1 | (G)11 | 11 | 4081 | 4091 |
| 3 | p6 | (CTTGCT)4 | 24 | 12951 | 12974 |
| 4 | p5 | (TGGCT)3 | 15 | 41915 | 41929 |
| 5 | p6 | (CTAACT)3 | 18 | 42337 | 42354 |
| 6 | p6 | (AGTTAG)3 | 18 | 42410 | 42427 |
| 7 | p1 | (T)15 | 15 | 47019 | 47033 |
| 8 | p5 | (AAAGC)3 | 15 | 53848 | 53862 |
| 9 | p1 | (A)10 | 10 | 54541 | 54550 |
| 10 | p4 | (CTTT)3 | 12 | 70819 | 70830 |
| 11 | p1 | (C)12 | 12 | 77767 | 77778 |
| 12 | p6 | (CTTGCT)3 | 18 | 80246 | 80263 |
| 13 | p1 | (T)10 | 10 | 81164 | 81173 |
| 14 | p1 | (T)10 | 10 | 91193 | 91202 |
| 15 | p6 | (GAACTT)3 | 18 | 98310 | 98327 |
| 16 | p1 | (A)10 | 10 | 99672 | 99681 |
| 17 | p6 | (AAGTTT)4 | 24 | 101065 | 101088 |
| 18 | p2 | (CT)5 | 10 | 106231 | 106240 |
| 19 | p6 | (AAGTTC)3 | 18 | 108920 | 108937 |
| 20 | p3 | (ATT)4 | 12 | 111733 | 111744 |
| 21 | p1 | (A)11 | 11 | 112451 | 112461 |
| 22 | p6 | (AAGTGT)4 | 24 | 119348 | 119371 |
| 23 | p1 | (A)10 | 10 | 119584 | 119593 |
| 24 | p1 | (A)10 | 10 | 121610 | 121619 |
| 25 | p6 | (GAACTT)4 | 24 | 122404 | 122427 |

**Continued Table 4 SSRs identified in the chloroplast genome in *Chloroidium* sp. W5**

| SSRs No. | type | SSRs | size | start | end |
| --- | --- | --- | --- | --- | --- |
| 26 | p4 | (CTTA)3 | 12 | 129593 | 129604 |
| 27 | p1 | (G)17 | 17 | 129887 | 129903 |
| 28 | p6 | (AAGTTC)4 | 24 | 134115 | 134138 |
| 29 | p1 | (C)12 | 12 | 138799 | 138810 |
| 30 | p1 | (A)10 | 10 | 140752 | 140761 |
| 31 | p2 | (AT)6 | 12 | 142682 | 142693 |
| 32 | p1 | (T)10 | 10 | 145272 | 145281 |
| 33 | p1 | (G)11 | 11 | 145284 | 145294 |
| 34 | p1 | (A)11 | 11 | 146008 | 146018 |
| 35 | p3 | (CAA)5 | 15 | 165761 | 165775 |
| 36 | p1 | (C)10 | 10 | 173140 | 173149 |
| 37 | p1 | (G)13 | 13 | 173159 | 173171 |
| 38 | p1 | (G)15 | 15 | 175756 | 175770 |
| 39 | p1 | (C)12 | 12 | 175991 | 176002 |
| 40 | p1 | (G)10 | 10 | 176654 | 176663 |

**Table 5 Tandem Repeat sequences identified in chloroplast genome of *Chloroidium* sp. W5**

| No. | position | | Period size | Copy number | Percent matches | Score | base composition | | | | Entropy (0-2) |
| --- | --- | --- | --- | --- | --- | --- | --- | --- | --- | --- | --- |
|  | Start | End |  |  |  |  | A | C | G | T |  |
| 1 | 3069 | 3110 | 14 | 3 | 100 | 84 | 50 | 14 | 35 | 0 | 1.43 |
| 2 | 6998 | 7071 | 10 | 7.4 | 96 | 139 | 29 | 50 | 9 | 10 | 1.69 |
| 1 | 4864 | 4889 | 2 | 13 | 100 | 0 | 52 | 23 | 23 | 30 | 23 |
| 2 | 6965 | 6993 | 1.9 | 15 | 100 | 0 | 58 | 37 | 13 | 41 | 6 |
| 3 | 12951 | 12976 | 4.3 | 6 | 100 | 0 | 52 | 0 | 34 | 15 | 50 |
| 4 | 30645 | 30679 | 1.9 | 18 | 88 | 0 | 52 | 34 | 11 | 17 | 37 |
| 5 | 33824 | 33937 | 5.4 | 21 | 100 | 0 | 228 | 34 | 9 | 33 | 22 |
| 6 | 40287 | 40351 | 2.4 | 27 | 72 | 10 | 69 | 27 | 21 | 23 | 27 |
| 7 | 49182 | 49218 | 2.1 | 18 | 94 | 0 | 65 | 18 | 2 | 5 | 72 |
| 8 | 50136 | 50177 | 2.8 | 15 | 81 | 0 | 57 | 52 | 16 | 28 | 2 |
| 9 | 56268 | 56302 | 2.4 | 15 | 90 | 4 | 54 | 51 | 20 | 28 | 0 |
| 10 | 60809 | 60848 | 2.1 | 19 | 90 | 0 | 62 | 22 | 12 | 27 | 37 |
| 11 | 61923 | 61994 | 2 | 36 | 97 | 0 | 135 | 26 | 27 | 26 | 19 |
| 12 | 71606 | 71639 | 2 | 17 | 100 | 0 | 68 | 41 | 11 | 35 | 11 |
| 13 | 81021 | 81049 | 1.9 | 15 | 100 | 0 | 58 | 27 | 6 | 17 | 48 |
| 14 | 88104 | 88148 | 2.1 | 21 | 87 | 0 | 63 | 28 | 17 | 28 | 24 |
| 15 | 101059 | 101093 | 5.8 | 6 | 96 | 0 | 61 | 34 | 2 | 17 | 45 |
| 16 | 107944 | 107979 | 2.4 | 15 | 95 | 0 | 63 | 11 | 36 | 13 | 38 |
| 17 | 108841 | 108891 | 3.4 | 15 | 88 | 0 | 84 | 5 | 33 | 17 | 43 |
| 18 | 119348 | 119372 | 4.2 | 6 | 100 | 0 | 50 | 36 | 0 | 32 | 32 |
| 19 | 122404 | 122439 | 6 | 6 | 93 | 0 | 63 | 33 | 19 | 16 | 30 |
| 20 | 124167 | 124197 | 2.1 | 15 | 93 | 0 | 53 | 0 | 29 | 19 | 51 |
| 21 | 134115 | 134161 | 7.8 | 6 | 92 | 0 | 76 | 34 | 10 | 17 | 38 |
| 22 | 161192 | 161239 | 2.3 | 21 | 89 | 3 | 80 | 25 | 29 | 29 | 16 |
| 23 | 170034 | 170079 | 2 | 23 | 95 | 0 | 83 | 23 | 26 | 19 | 30 |

**Table 6 Codon usage in chloroplast genome of *Chloroidium* sp. W5**

| Codon | Frequency | RSCU | AA | Codon | Frequency | RSCU | AA |
| --- | --- | --- | --- | --- | --- | --- | --- |
| UUU(F) | 723 | 1.35 | Phe | UAU(Y) | 383 | 1.38 | Tyr |
| UUC(F) | 348 | 0.65 | Phe | UAC(Y) | 169 | 0.61 | Tyr |
| UUA(L) | 878 | 2.29 | Leu | UAA(*) | 34 | 2.04 | \| |
| UUG(L) | 334 | 0.87 | Leu | UAG(*) | 11 | 0.66 | \| |
| CUU(L) | 524 | 1.37 | Leu | CAU(H) | 265 | 1.24 | His |
| CUC(L) | 166 | 0.43 | Leu | CAC(H) | 164 | 0.76 | His |
| CUA(L) | 280 | 0.73 | Leu | CAA(Q) | 732 | 1.58 | Gln |
| CUG(L) | 119 | 0.31 | Leu | CAG(Q) | 192 | 0.42 | Gln |
| AUU(I) | 702 | 1.77 | Ile | AAU(N) | 486 | 1.18 | Asn |
| AUC(I) | 207 | 0.52 | Ile | AAC(N) | 340 | 0.82 | Asn |
| AUA(I) | 282 | 0.71 | Ile | AAA(K) | 962 | 1.53 | Lys |
| AUG(M) | 342 | 1 | Met | AAG(K) | 296 | 0.47 | Lys |
| GUU(V) | 618 | 1.80 | Val | GAU(D) | 575 | 1.39 | Asp |
| GUC(V) | 134 | 0.39 | Val | GAC(D) | 255 | 0.61 | Asp |
| GUA(V) | 434 | 1.26 | Val | GAA(E) | 760 | 1.28 | Glu |
| GUG(V) | 190 | 0.55 | Val | GAG(E) | 425 | 0.72 | Glu |
| UCU(S) | 449 | 1.54 | Ser | UGU(C) | 140 | 1.37 | Cys |
| UCC(S) | 116 | 0.40 | Ser | UGC(C) | 64 | 0.63 | Cys |
| UCA(S) | 352 | 1.20 | Ser | UGA(*) | 5 | 0.3 | \| |
| UCG(S) | 230 | 1.79 | Ser | UGG(W) | 264 | 1 | Trp |
| CCU(P) | 335 | 1.32 | Pro | CGU(R) | 344 | 1.48 | Arg |
| CCC(P) | 128 | 0.50 | Pro | CGC(R) | 186 | 0.80 | Arg |
| CCA(P) | 376 | 1.48 | Pro | CGA(R) | 376 | 1.62 | Arg |
| CCG(P) | 176 | 0.69 | Pro | CGG(R) | 122 | 0.53 | Arg |
| ACU(T) | 432 | 1.39 | Thr | AGU(S) | 344 | 1.18 | Ser |
| ACC(T) | 206 | 0.66 | Thr | AGC(S) | 263 | 0.90 | Ser |
| ACA(T) | 449 | 1.44 | Thr | AGA(R) | 288 | 1.24 | Arg |
| ACG(T) | 160 | 0.51 | Thr | AGG(R) | 79 | 0.34 | Arg |
| GCU(A) | 695 | 1.74 | Ala | GGU(G) | 653 | 1.72 | Gly |
| GCC(A) | 216 | 0.54 | Ala | GGC(G) | 228 | 0.60 | Gly |

**Continued Table 6 Codon usage in chloroplast genome of *Chloroidium* sp. W5**

| Codon | Frequency | RSCU | AA | Codon | Frequency | RSCU | AA |
| --- | --- | --- | --- | --- | --- | --- | --- |
| GCA(A) | 471 | 1.18 | Ala | GGA(G) | 373 | 0.98 | Gly |
| GCG(A) | 215 | 0.54 | Ala | GGG(G) | 264 | 0.70 | Gly |

Note: RSCU: relative synonymous codon usage.

**
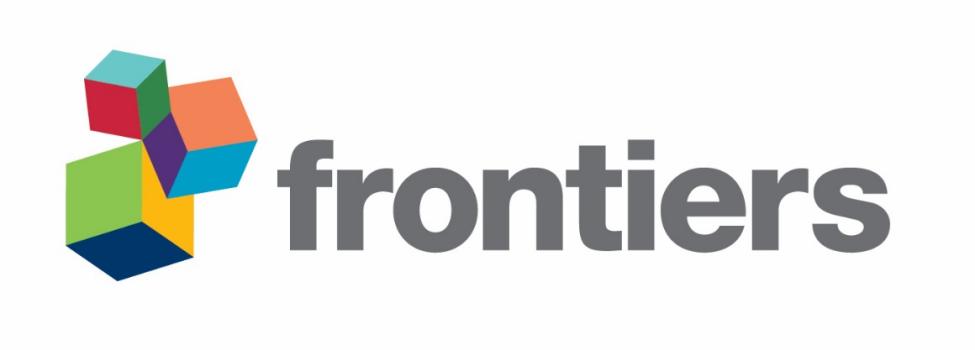
**
